# Assessment of static posturography and pedobarography for the detection of unilateral forelimb lameness in ponies

**DOI:** 10.1186/s12917-018-1462-8

**Published:** 2018-05-02

**Authors:** Lidia Pitti, Maarten Oosterlinck, Maria L. Díaz-Bertrana, José M. Carrillo, Mónica Rubio, Joaquin Sopena, Angelo Santana, José M. Vilar

**Affiliations:** 1 Departamento de Patología Animal, Universidad de Las Palmas de Gran Canaria, Las Palmas de Gran Canaria, Spain; 2 Department of Surgery and Anaesthesiology of Domestic Animals, Faculty of Veterinary Medicine, Ghent University, Ghent, Belgium; 3 Departamento Medicina y Cirugía Animal, Cátedra García Cugat, Universidad CEU Cardenal Herrera, Valencia, Spain; 4 Departamento de Matemáticas, Universidad de las Palmas de Gran Canaria, Las Palmas, Spain; 5 Departamento de Patología Animal, Instituto Universitario de Investigaciones Biomédicas Y Sanitarias, Universidad de Las Palmas de Gran Canaria, Arucas, Spain

**Keywords:** Balance, Center of pressure, Postural sway, Stabilography, Posturography, Pedobarography, Pony

## Abstract

**Background:**

Static posturography and pedobarography are based on the detection of postural imbalance and, consequently, the pressure redistribution between limbs in lame subjects. These techniques have proven to be useful for the detection of lameness in humans and dogs. The main objective of this study was to test the suitability of static posturography and pedobarography in diagnosing lameness in ponies.

A pressure platform was used to obtain postural data (statokinesiograms, mean X and Y, length, LFS ratio, and mean velocity) from 10 sound ponies and 7 ponies with unilateral forelimb lameness. Static pedobarographic data (pressure distribution, mean pressure, and peak pressure) were also collected and compared with force plate data (peak vertical force and vertical impulse) obtained from the same animals at the walk.

**Results:**

Significant differences were seen between lame and sound ponies for almost all evaluated parameters. With this sample size, differences between lame and sound limbs/groups were detected with a statistical power of 90%, except for mean X and Y.

**Conclusions:**

Static posturography and pedobarography provide a complementary approach for lameness detection in equids.

## Background

To overcome the inherent limitations of a subjective visual evaluation of lameness, kinetic [1, 2] and kinematic [3, 4] analyses have been introduced in equine veterinary medicine. Force platforms are considered the “gold standard” in the evaluation of lameness, and inertial sensor and optical motion capture systems have recently become commercially available [5]. However, the installation of a force platform or an optical motion capture system is technically and financially demanding, precluding widespread use outside of highly specialized laboratories or hospitals [6].

Alternatively, pressure platforms may provide a more practical alternative to force plate analysis. This equipment is portable, rather inexpensive, and provides not only information on ground reaction forces (GRF) (e.g. peak vertical force (PVF) and vertical impulses (VI)), but also pressure-related (i.e., pedobarographic (PB)) parameters as mean or peak pressures (MP, PP, respectively) [7–10].

In equine veterinary medicine, pressure plates have been used for studying hoof landing patterns and unrollment [11], effects of trimming [12], and symmetry and hoof balance in sound horses and ponies [13, 14].

A new approach to lameness detection in the veterinary medicine is based in the detection of postural (i.e., posturographic (PT)) characteristics determined by a number of body center of pressure (COP) path parameters as area, length, velocity, etc. Changes in these characteristics obtained from force plate analysis have been used to assess the development of postural balance in foals [15], the postural effects of administering detomidine® [16] and blindfolding in healthy horses [17]. Moreover, it has been suggested that COP analysis could have diagnostic value in differentiating lame from ataxic horses [18]. Regarding lameness, it has been shown in lame subjects that a load transfer towards the sound limbs occurs [19, 20], although this redistribution pattern could vary depending on a horse’s gait [21]. Consequently, lameness would also affect static COP path characteristics and pressure values (MP, PP, etc.). This has been proven in dogs in which this technique was effective for diagnosing lameness and assessing the effect of treatments for osteoarthritis [22].

A unique advantage of this new technique for assessing lameness is that measurements are taken with the animal standing still; thus, data can be collected in relatively small spaces [23].

Based on widespread application in humans, the most frequently used PT parameters for the diagnosis of postural alterations are as follows: (1) statokinesiograms, defined as the area determined by an ellipse that contains 90% of the recorded points of the COP trajectory [24], measured in mm^2^, and a smaller area is associated with superior stability [22, 25]; (2) mean COP X and Y (mm), which quantifies the mediolateral and craniocaudal COP displacements independently, and, similar to statokinesiograms, smaller displacement is associated with better stability [25, 26]; (3) COP length (m), which is also called total path length [24], is the length of the line that joins the recorded points of the COP trajectory, where a higher value means more instability [27]; (4) LFS (length in function of surface), which is defined as the correlation coefficient between the COP length and its surface. This parameter provides information about the accuracy of postural control and the effort made by the subject [28], which increases its value [29]; (5) mean velocity (mm/s) of COP sway increases with instability, and this parameter may be one of the most accurate variables for the assessment of postural stability [30, 31].

Secondly, main PB data have (1) static pressure distribution expressed as a percentage (%) of total pressure exerted by each limb [22] and (2) mean pressure (MP) and peak pressure (PP) of limbs detected by the activated platform sensors [32] measured in kilopascals.

We hypothesized that static PT and PB parameters may be valid to detect lameness in equids. Therefore, the aim of this study was to test the appropriateness of a set of static PT and PB parameters in lameness detection in ponies.

## Methods

### Animals

The study sample consisted of 17 unshod ponies of similar conformation used for pleasure riding by children. The animals’ hooves were trimmed 1 week before the study. All ponies were examined and judged by an experienced veterinary clinician (LP) under AAEP criteria, i.e., absence of lameness in the medical history of the pony, visual and hands-on exams, application of hoof testers to the hooves, joint flexion tests and evaluation of ponies in motion (walk and trot). Lameness was graded according to the AAEP scoring system (0–5). In the case of lameness, further examination included diagnostic anesthesia and dedicated imaging in order to identify the cause of lameness.

Ten of the 17 ponies were judged to be clinically sound (AAEP score 0) and, consequently, were considered part of the control group. Age ranged from 5 to 13 years; body mass from 174 to 180 kg.. On the other hand, 7 ponies presented forelimb lameness (grade 2–3/5 AAEP) attributable to desmitis of branches of the suspensory ligament (*n* = 3), acute tendinopathy of the superficial digital flexor tendon (*n* = 1), and fetlock osteoarthritis (n = 3). Age ranged from 9 to 15 years; body mass from 167 to 193 kg.

### PT and PB study

A pressure platform (EPS/R1, Loran Engineering, Bologne, Italy) with Biomech software (Loran Engineering, Bologne, Italy) was used. The device contained a total of 2304 pressure sensors (density 1 sensor/cm^2^) distributed in an area of 48 × 48 cm, with an acquisition frequency of 100 Hz and a measuring range of 30–400 Kpa. The platform was placed in a purpose-built cavity to maintain it leveled with the floor. All the support surface, included the platform, was covered with a flexible leatherette mat of 2 mm thick; in this way, fore and hindlimbs were at the same level.

Animals were placed with both forelimbs on the platform while standing still for at least 20 s. A total of three trials were obtained from each animal. A trial was considered valid when no movement of the limbs, head, and/or neck was observed, and the handler did not had to have any physical contact to restraint the animal during the recording. The obtained PT data included statokinesiograms, Mean COP X and Y (mm), COP length (mm), LFS ratio, and mean COP velocity (mm/s). The obtained PB data included static pressure distribution, MP, and PP (Kpa).

### Force platform analysis

The differences in static PB parameters (between contralateral limbs and between groups) obtained with the pressure platform were compared with the differences in PVF and VI obtained at a walk from the same animals with a 4-sensors force platform of 35 × 35 cm and 250 Hz of sample frequency (Pasco, California, USA) placed adjacently to the pressure platform. DataStudio software (Pasco, California, USA) was used to obtain PVF (N) and VI (Ns) values from three valid trials. A trial was considered valid when ponies walked over the platform at a velocity range of 1.6 ± 0.3 m/s and had an acceleration of ≤0.3 m/s^2^. These parameters were obtained by using a motion sensor (Pasco, California, USA).

PVF and VI mean values were normalized to body weight (% BW).

For comparison purposes, limbs with lower forces than the contralateral limb were considered as ‘lame’ limbs (LL), whereas the other limb was considered as ‘control’ limb (CL) in both groups. The difference in percentage between CL and LL was calculated using the following formula: Δ% = 200* (CL-LL)/(CL + LL) [33].

### Statistical analysis

A linear mixed effects model was used for the analysis of data, using the following formula:

$$ {y}_{ij}={\beta}_i+{b}_i+{\epsilon}_{ij} $$

where *y*_*i*_*j* is the value of the response variable in the j^th^ measure made under status *i* (sound/lame).

Ninety-five percent confidence intervals (95% CI) were calculated for the model parameters and the differences between groups. Normality in the residuals was checked using the Shapiro-Wilk test. Homoscedasticity of the residuals was checked by the Levene test. For all tests, a significance level of 5% was used. The power of the statistical tests was evaluated by whether the estimates of the variances obtained in the model fit. For every test, we have calculated which difference value could be detected with a power of 90%. Statistical analysis was performed with ‘R’ statistical language and environment, version 3.3.2. (https://www.R-project.org/).

## Results

Mean values of age (mean ± SD) were 11.61 ± 4.47 years; body mass 174 ± 6.31 kg, and height at the withers 1.20 ± 0.05 m. There was no statistically significant difference in the body mass of ponies in the control group vs. the study group (*P* = 0.32).

The mean (± SD) values and 95% CI of all obtained PT and PB parameters are shown in Tables 1 and 2, respectively. All data were normally distributed and homoscedastic (*p* ≥ 0.06 and *p* ≥ 0.07, respectively). Detectable differences with a statistical power of 90% are shown. The sample size used in this study (7 lame and 10 control ponies with three trials each) proved to be large enough for detecting significant differences, consisting of 90% statistical power for almost all variables.

**Table 1:** Mean ± SD, 95% confidence interval and difference between study and control groups for PT parameters. The 90% statistical power value when significant differences were found is also provided

Statokinesiogram (mm^2^)		Difference	∆ 90%
Study			
	35.73 ± 19.61		
	24.85, 46.61		
Controls			
	3.33 ± 2.53	32.40 ± 6.30	22.60
	−5.63, 12.29	18.97, 45.84	
Mean X (mm)			
Study			
	1.33 ± 0.57		
	0.93, 1.72		
Controls			
	0.82 ± 0.41	0.51 ± 0.23	0.82^a^
	0.49, 1.15	0.02, 1.00	
Mean Y (mm)			
Study			
	0.43 ± 0.20		
	0.32, 0.55		
Controls			
	0.44 ± 0.25	0.01 ± 0.07	
	0.34, 0.55	0.01, 0.07	
Lenght (mm)			
Study			
	102.66 ± 39.98		
	81.36, 123.95		
Controls			
	46.08 ± 20.52	56.58 ± 11.53	34.49
	31.55, 60.60	32.01, 81.15	
LFS			
Study			
	32.44 ± 29.65		
	17.85, 47.02		
Controls			
	6.73 ± 4.03	25.70 ± 6.60	5.56
	4.39, 9.07	11.63, 39.78	
Mean V (mm/s)			
Study			
	6.09 ± 1.92		
	4.99, 7.18		
Controls			
	2.38 ± 1.12	3.70 ± 0.59	1.78
	1.65, 3.11	2.45, 4.96	

Difference with a 90% power value in Mean X (^a^) is higher than detected difference; this means that the parameter is unable to distinguish between lame and sound ponies

**Table 2:** Mean ± SD, 95% confidence interval, and % difference between CL and LL limbs in both study and control groups for PB parameters. The 90% statistical power value when significant differences were found is also provided

	LL	CL	% Difference	∆ 90%
Pressure distribution				
Study				1.2
	42.82 ± 1.49%	57.18 ± 1.49%	7.18 ± 1.49^a^	
	42.09, 43.55	56.45, 57.91	6.48, 7.88	
Controls				
	49.44 ± 3.32%	50.56 ± 3.32%	0.56 ± 3.32^a^	
	48.09, 50.80	49.20, 51.91	−0.84, 1.96	
MP				
Study				12.2
	83.23 ± 10.27	157.54 ± 17.50	47.2 ± 4.37^b^	
	77.86, 88.60	148.68, 166.39	41.67, 52.80	
Controls				
	109.33 ± 17.97	111.42 ± 18.33	1.9 ± 1.98^b^	
	97.78, 120.89	99.67, 123.18	1.70, 5.4	
PP				
Study				5.4
	310.73 ± 49.35	398.59 ± 18.14	22 ± 11.76^b^	
	284.39, 337.07	387.96, 409.22	16.07, 28.07	
Controls				
	335.44 ± 32.33	357.61 ± 63.19	6.02 ± 11.23^b^	
	316.34, 354.54	329.49, 385.73	0.10, 12.53	
PVF				
Study				11.8
	66.84 ± 6.94	91.01 ± 6.67	26.6 ± 1.26^b^	
	61.66, 72.02	86.06, 95.96	21.60, 26.74	
Controls				
	72.58 ± 3.53	72.80 ± 3.27	0.3 ± 0.86^b^	
	71.10, 74.06	71.41, 74.18	−1.95, 1.52	
VI				
Study				14.0
	31.05 ± 2.91	40.38 ± 4.93	23.1 ± 1.06^b^	
	28.98, 33.11	37.60, 43.17	17.95, 28.75	
Controls				
	35.48 ± 2.56	35.79 ± 2.65	0.9 ± 0.56^b^	
	34.11, 36.86	34.45, 37.14	−0.81, 1.43	

^a^In regards to the ideal symmetry (i.e., 50% for each limb); ^b^In regards to CL value

### PT data

**Fig. 1:**
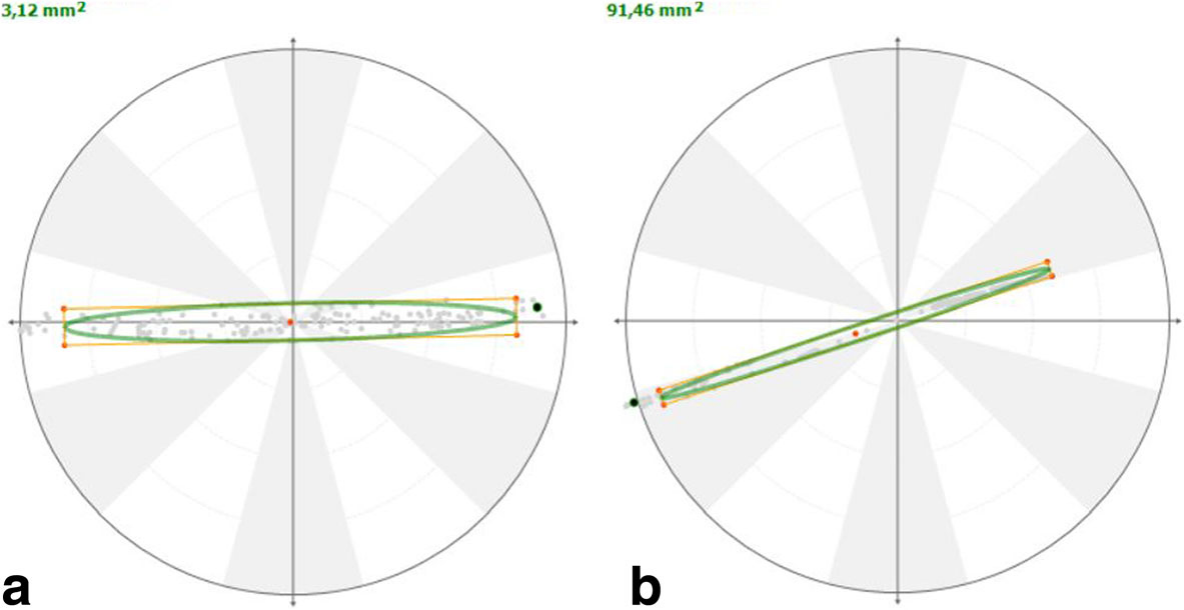
Statokinesiograms obtained from a sound pony (**a**) and a pony with a right fore lameness (**b**). Compared with the sound animal, the ellipse in the lame pony is asymmetrically displaced to the left side due to the body COP being shifted to the left more frequently than to the right side. In addition, the area of the ellipse is much greater (3.12 mm^2^ in the sound pony vs. 91.46 in the lame pony)

**Fig. 2:**
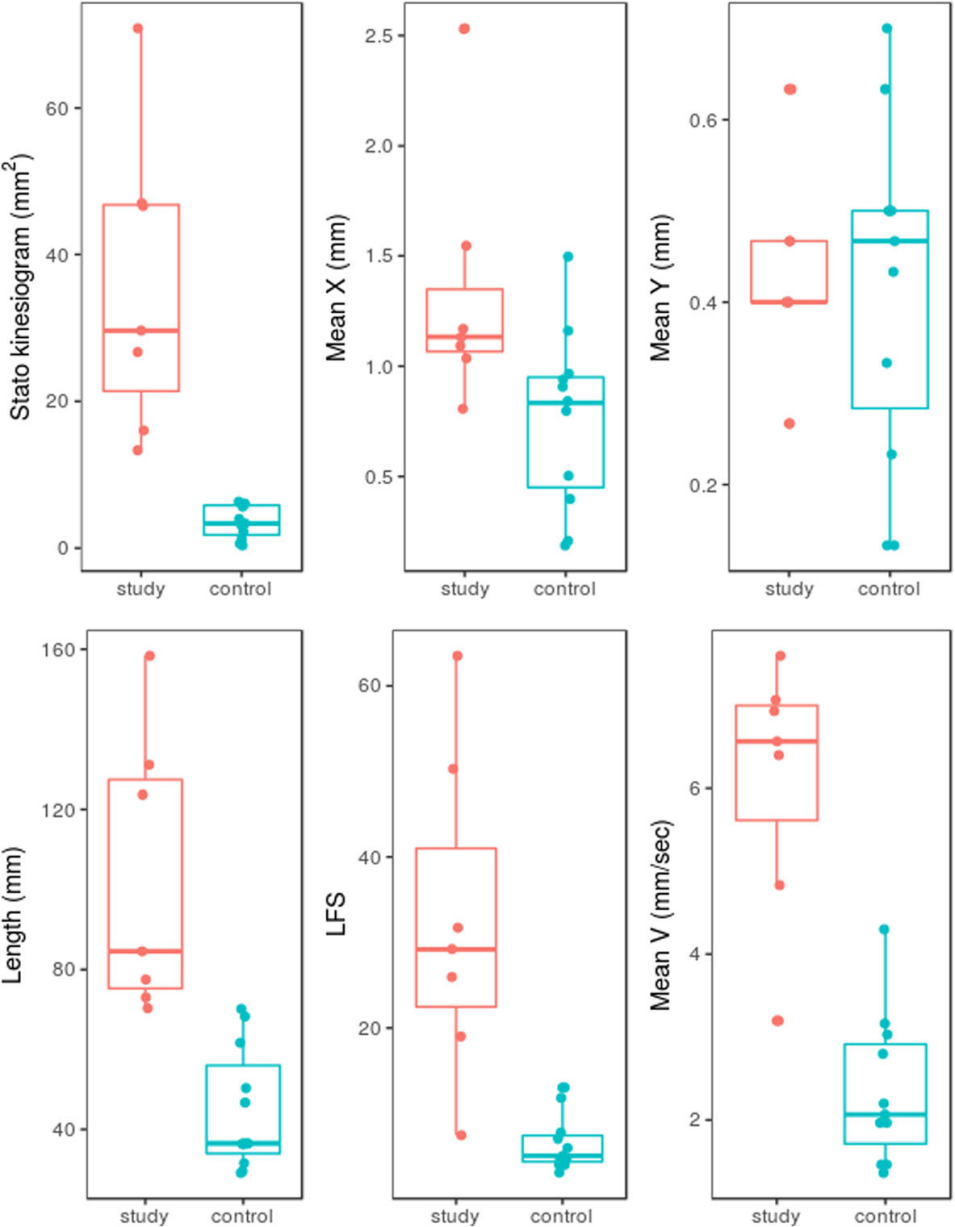
Boxplots of the PT parameters. Except for Mean Y, all values in the lame group are significantly larger than in the sound group

Data from statokinesiograms (Fig. 1a, b), Mean X, length, LFS, and mean COP velocity showed significantly higher values (*p* ≤ 0.03) in lame ponies compared with the control group, which is compatible with a higher COP sway (i.e. instability in the lame animals). Mean X failed to reach the required statistical power. Mean Y showed no significant differences between groups (*p* = 0.88) (Fig. 2).

### PB data

**Fig. 3:**
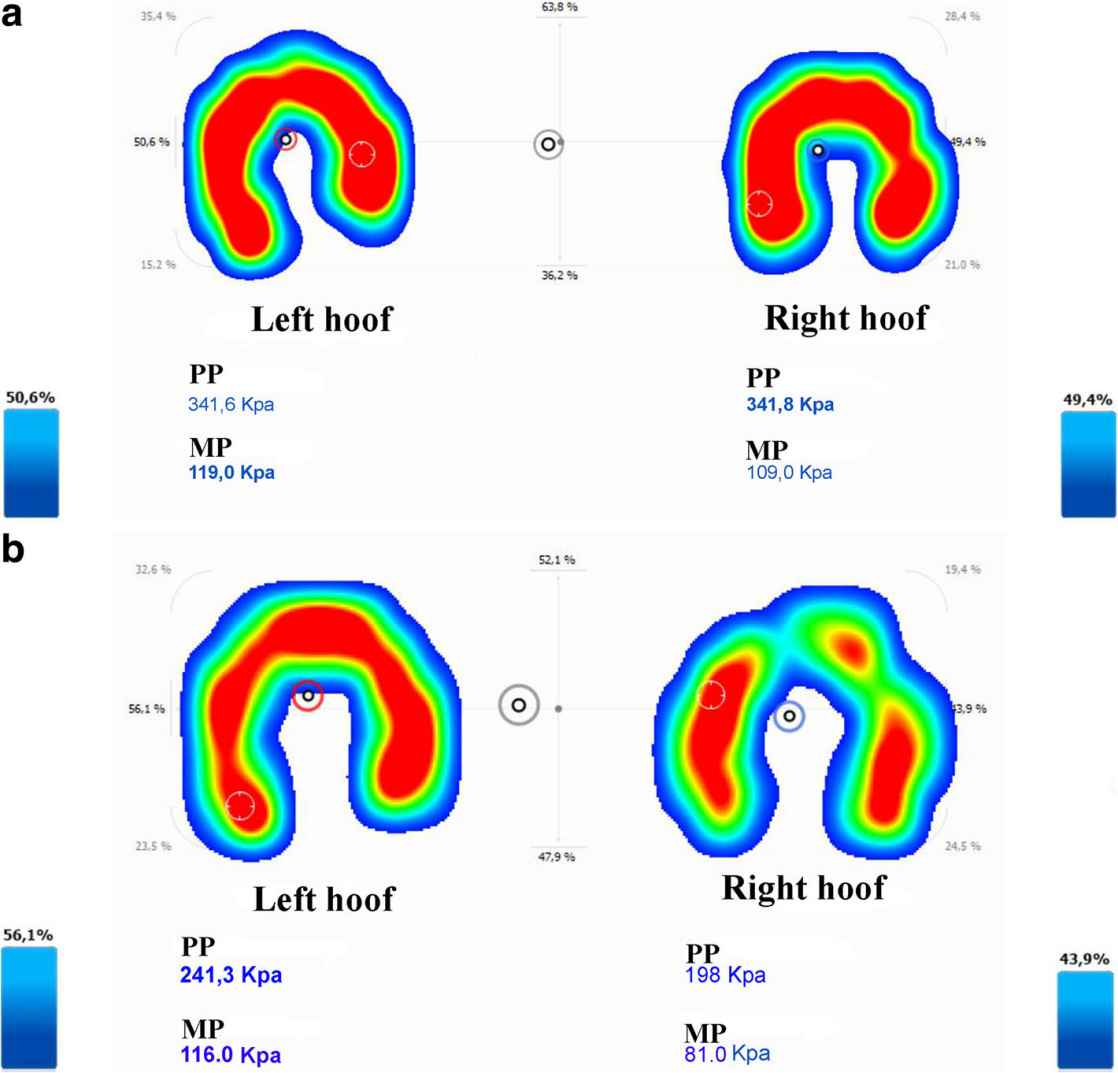
Pedobarography in a sound pony (**a**) and in a pony with a right fore lameness (**b**). The differences in pressure distribution, MP, and PP between the left and right hooves are much higher in the lame group (**b**) than in the control group (**a**). A left displacement of the body COP can be also seen (black/grey circles)

Differences in static pressure distribution (Fig. 3), MP and PP values between LL and CL showed a significant difference in the lame group (*p* ≤ 0.023), which is in contrast with the data from the control group (*p* ≥ 0.053). Compared with the sound group, CL values were significantly higher in the lame group for all measured parameters, which proves a pressure redistribution to the sound contralateral limb in lame ponies.

### Force platform data

**Fig. 4:**
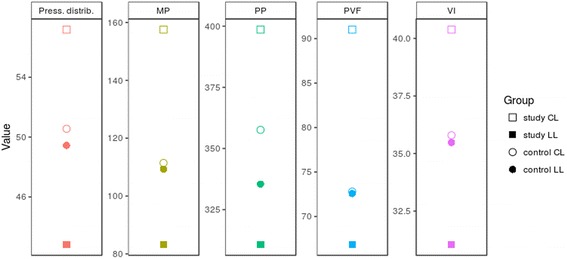
Comparison of differences between contralateral PB parameters with force plate variables (PVF and VI), visually illustrating greater differences between LL and CL in the study group than in the control group of sound ponies

In agreement with the PB data, PVF and VI values showed significant differences between LL and CL in the lame group when compared with the control group (*p* ≤ 0.0001) (Fig. 4). All PB and force platform data reach the 90% statistical power.

## Discussion

Building further on previous reports detailing stabilographic variables in sound horses [23], this study is the first to describe static PT and PB data for the detection of lameness in equids, specifically in ponies.

In the present study, almost all parameters proved suitable to detect lameness, similar to what has been described in humans [24] and dogs [22]. Among these variables, statokinesiograms provide the most reliable information, which confirms other reports [31]. These similarities may be explained because this value corresponds with the ellipse area containing 90% of COP sway points, while discarding the other 10% usually corresponding to unavoidable head tilts and nods.

Statokinesiogram values in lame ponies were similar to those reported in dogs, where values in cases of unilateral elbow dysplasia were under 45 mm^2^ [22]. Surprisingly, these values are lower than those considered normal in humans (≤ 100 mm^2^) [34]. This is in agreement with previous results obtained in horses [23] and is probably related to the four limbs providing a larger base of support than in a bipedal situation [18].

Another surprising finding was that in sound ponies latero-lateral sway was much bigger compared with that cranio-caudal, in contrast to humans, where predominant COP sway is the anteroposterior axis [35]. The greater distance between ipsilateral limbs and contralateral limbs provides more stability in the cranio-caudal sense, as previously reported in horses [17, 18]. This could also explain why the only parameter that did not show statistical differences between sound and lame ponies was the Mean Y.

A statistically significant difference between lame and sound groups was found for Mean X; however, this difference was very small (less than 1 mm) and could not be detected with 90% statistical power. For this reason, we believe that the minimal differences observed in this parameter may be irrelevant and render it not as reliable as the other variables to detect mild lameness.

Previous studies in dogs have suggested the suitability of the Area of support for the detection of lameness [10] as paws expand, although not linearly, depending of the applied pressure [22]. However, the relative rigidity of the equine hoof when compared with dog pads do not allow for the detection of acute changes in contact area. Until now, it has only been found useful to measure hoof size asymmetry, which can be observed, for example, in chronically lame limbs [36].

Notwithstanding their gold standard status in gait analysis [5], force plate analysis of GRF is limited to highly specialized labs or hospitals. This is not only because the installation is complicated and expensive, but also because gathering data is time-consuming and involves repeating many trials to record data of individual limbs. Moreover, trial repetition implies variations in velocity, which should be minimized. In contrast, static PT and PB analysis only requires a portable and rather inexpensive pressure platform and a minimum space to maintain the animal standing still, without the need to control speed.

However, in the veterinary field, static PT and PB studies have a main disadvantage in that, compared with humans, it is more difficult to maintain an animal completely immobile during a longer time. In humans, this time period has been classically set between 30 and 52 s [31, 34, 37]. In our study, we used a recording time of 20 s, which is an intermediate value compared with other postural equine studies [15, 17, 23, 25]. In our experience, extending the measurement beyond 20 s created problems because of animal movements, since the aim was to maintain the required steady position. It is important to note that this technique may be impossible to perform in certain nervous or fractious animals.

There are threelimitations of this study. First, as stated above, compensatory pressure redistribution in lame horses implies not only the contralateral limb [33, 38]; thus, it would be interesting to include data from hindlimbs in a subsequent study as well as determination if correlation exist between PT and PB values with lameness degree. Unfortunately, the relative small dimensions of the pressure platform impede the simultaneous analysis of more than two limbs. Second, direct comparison with previous studies using force or pressure platform equipment should be considered with caution, since different technology may alter the results [23]. Third, and last, besides its application for detecting lameness, this new technique may also serve for the detection of postural imbalances caused by neurological disorders as in humans, although further studies are required to distinguish between lame from ataxic animals. Should be also interesting to know what kind of correlation exist between our PB parameters in static animals with those obtained from ponies at walk or trot.

## Conclusions

This study proves that static PT and PB parameters can be useful tools for the detection of equine lameness, especially in ponies.

## Abbreviations

CL: Sound limb in the study group or the limb with the higher value in the control group
COP: Center of pressure
GRF: Ground reaction forces
LFS: Length/area ratio
LL: Lame limb in the study groups or limb with lesser value in the control group
MP: Mean pressure
PB: Pedobarography
PP: Peak pressure
PT: Posturography
PVF: Peak vertical force
VI: Vertical impulse
